# Should we be concerned when the anterior approach to the hip goes accidentally medial? A retrospective study

**DOI:** 10.1186/s42836-024-00269-9

**Published:** 2024-09-01

**Authors:** Giuseppe Geraci, Alberto Di Martino, Niccolò Stefanini, Matteo Brunello, Federico Ruta, Federico Pilla, Francesco Traina, Cesare Faldini

**Affiliations:** 1 1st Orthopaedic and Traumatologic Department, IRCCS Rizzoli Orthopedic Institute, Via G.C. Pupilli 1, Bologna, 40136 Italy; 2 Department of Biomedical and Neuromotor Science-DIBINEM, University of Bologna, 40136 Bologna, Italy

**Keywords:** Total hip arthroplasty, Direct anterior approach, THA, DAA, Proximal femur, Acetabular component, Femural component

## Abstract

**Background:**

The direct anterior approach is increasingly used for primary total hip arthroplasty (THA) due to its minimally invasive nature and rapid recovery time. Difficulties in identifying the correct intermuscular interval can arise during the procedure, sometimes resulting in excessive medial exposure. This study aimed to evaluate demographics and risk factors, outcomes, and potential complications in those THA patients in which a medialized approach was performed.

**Methods:**

We retrospectively reviewed cases of anterior THA to identify cases where the surgical approach to the hip was more medial than the standard interval. Demographic data, operative time, blood loss, intraoperative and postoperative complications, radiographic findings were collected and compared with a control group of 50 THA performed using the standard anterior intermuscular interval.

**Results:**

In a series of 1,450 anterior total hip arthroplasty (THA) procedures performed between January 2018 and December 2021, with an average follow-up of 33 ± 22.3 months, six patients (0.4%) had a medialized surgical interval. In one case the superficial layer was medial to the sartorious muscle while in the other five cases, the interval was lateral to the sartorius superficially, and medial to the rectus femoris deeply. Four out of 6 patients (66.6%) showed neuropraxia affecting the femoral nerve, and 3 out of 6 (50%) had involvement of the lateral femoral cutaneous nerve. In 6 out of 6 patients (100%), surgery was performed during the learning curve of DAA. No patients in the control group developed femoral nerve neuropraxia, and 2 out of 50 patients (4%) showed involvement of the lateral femoral cutaneous nerve.

**Discussion and conclusion:**

The anterior approach can rarely result in excessive medial exposure to the hip joint, especially during the learning curve. In our study cohort, an increased rate of neurological complications and reduced outcomes were observed, thereby rendering this event of particular clinical significance. To avoid unconventional intermuscular intervals, patient positioning and correct identification of the muscle bellies by recognizing the orientation of the muscle fibers are useful, together with the identification and ligation of the circumflex vessels, to ensure the identification of the correct intermuscular interval.

## Background

The direct anterior approach (DAA) is increasingly used for total hip arthroplasty (THA) for its advantages in terms of low muscle damage, early recovery enhancement, potential reduction of hospital length of stay, and improved surgery-related patient satisfaction [1–6]. DAA uses the intermuscular and internervous interval between the sartorius and the tensor fasciae latae (TFL) superficially and between the gluteus medius and rectus femoris (RF) deeply [7]. This interval allows for a wide and safe exposure of the acetabulum and proximal femur, enabling adequate positioning of implant components with minimal muscle damage and reduced bleeding [8, 9]. The approach is increasingly used both in high-volume centers and in small healthcare facilities because it may reduce hospital length of stay, contributing to successful enhanced recovery after surgery (ERAS) protocols [10, 11]. However, DAA is a demanding technique compared to other approaches, with a steep learning curve and potentially higher rates of intraoperative complications [12, 13]. In particular, high percentages of femoral fractures and implant malpositioning were reported, leading to potential mechanical failure and need for revision surgery [14, 15].

In clinical practice, complications may arise when the correct surgical interval is not utilized. In general, a surgical interval provides a pathway that allows the surgeon to access the area of interest, such as the joint or bone, while avoiding critical neurovascular structures, providing the best possible exposure to the operative field. Correct identification of the interval ensures that the procedure adheres to the principles of atraumatic surgery. This precision is essential for reducing postoperative pain, minimizing the risk of complications, and promoting a faster recovery.

**Fig. 1:**
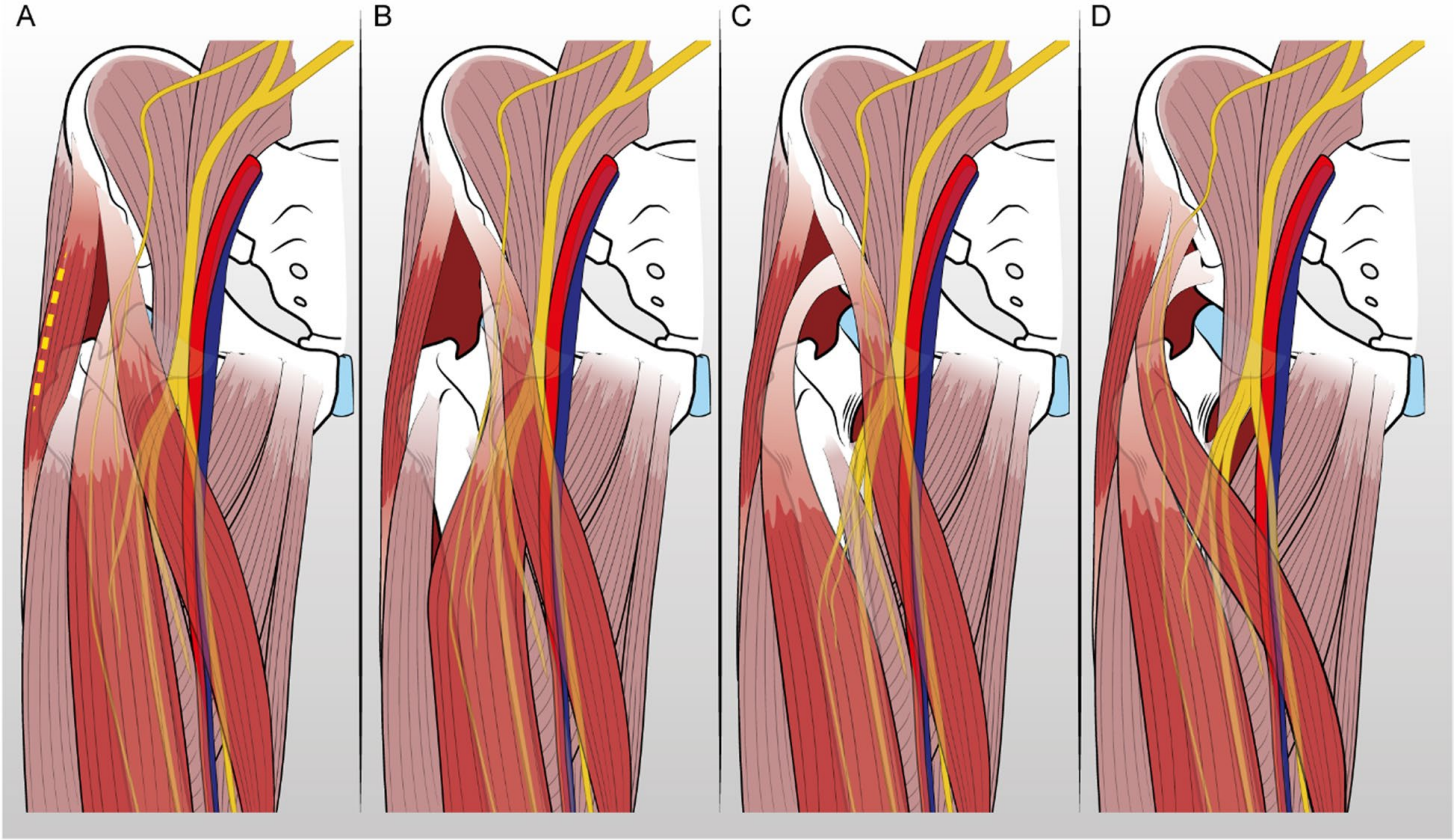
Normal anatomy of the anterior region of the hip joint (**A**), standard anterior approach to the hip (**B**), anterior approach medial to the Rectus Femoris (**C**), anterior approach medial to both the Rectus Femoris and the Sartorius (**D**)

Difficulties in identifying the correct intermuscular interval can arise during DAA THA, sometimes resulting in excessive medial exposure of the joint through an unconventional muscular interval (Fig. 1). Deviations from the standard approach, if not promptly recognized and corrected, are deemed responsible for several complications, including impaired exposure and visualization, protracted surgical time, increased risk of neurovascular injury, and inadequate prosthetic component positioning, although specific data are absent in the current scientific literature.

To improve knowledge on this topic, the current manuscript aimed to present a case series in which the surgical approach to the hip was accidentally more medial than the original DAA. Patients were retrieved out of a cohort of patients from a high-volume DAA THA centre and analyses of patients’ demographics, risk factors, main outcomes, and potential complications were presented. To the authors' knowledge, this was the first study to inform and warn surgeons about this challenging pitfall, and to evaluate related outcomes and complications.

## Materials and methods

The current study and all the case collections were approved by the Local Ethical Committee (CE-AVEC) with the code 021 ANT-HIP, 347/2021/Oss/IOR. Patients in the study group were retrospectively identified through a review of surgical records of primary THAs performed from January 2018 to December 2021 in a single surgical unit at the authors' institution. Hip surgery unit was composed of 2 senior surgeons, 5 consultants, and 6 fellows. Each year, 600 to 800 DAA primary THAs are performed. It is a standard practice for the procedure to be initiated by the consultant/fellow/resident, with the definitive implant placement performed by the senior surgeons or under their supervision. According to the surgical reports and team meeting transcripts, when the senior surgeon or consultant realized that a medialized surgical approach was used, a report was prepared, and these patients were identified and collected for this study. Hip surgeons in the surgical unit were then queried to identify any additional cases.

Only patients undergoing THA for primary hip osteoarthritis (OA) with evidence of an unconventional anterior approach were included in the study group. Exclusion criteria were THA in patients with secondary OA, previous hip surgeries, such as femoral or acetabular osteotomy for deformity correction, severe deformities, and connective tissue disorders. Patients operated on for THA for medial neck femur fractures for trauma or tumor were also excluded.

Furthermore, a control group was formed by including 50 consecutive patients who underwent THA by DAA, beginning January 2020 (to achieve a comparable average follow-up of the two groups). All participants had been followed up until December 31, 2023. The same exclusion criteria were applied to the control group.

Surgery consisted of THA by DAA using a traction table and all implants were cementless. In brief, a modified mini-invasive Heuter approach modified by Faldini was used [7]. Skin incision was approximately 8 cm long, starting 2 cm lateral and distal anterior superior iliac spine with the leg positioned in a slight flexion and internal rotation, with the patella in a neutral position. After the dissection of the skin and subcutaneous tissues, the fascial plane was reached. The incision of the fascia was made over the muscular belly of the TFL, and then a blunt dissection was performed to create the interval between the TFL and sartorius. Subsequently, the rectus femoris was medially retracted, and the lateral circumflex vessels were identified and ligated. This was followed by the exposure of the capsule and a V capsulotomy followed by femoral neck osteotomy, head removal, and acetabular cup preparation. After cup impaction and liner positioning, the proximal femur was exposed and broached, and the stem was impacted. Head positioning and joint reduction followed.

Demographic data (age at surgery, sex, BMI), intraoperative data and blood loss (decrease in hemoglobin values after surgery—ΔHb), clinical outcomes (preoperative and postoperative Harris Hip Score—HHS), intraoperative and postoperative complications, radiographic findings (positioning of prosthetic components in coronal supine radiographs: abduction of acetabular cup and coronal alignment of the stem) of the study group were collected and compared. Medical records and surgical reports were retrospectively reviewed, and lead surgeons were interviewed to determine at what point during the procedure they realized to be in the unconventional muscular interval. The number of previous DAA THA surgeries and average THA procedures per year were noted for each surgeon at the time of the index surgical procedure.

Data were described with the use of descriptive statistics. Due to the small number of cases in the study group, statistical analyses were performed only when deemed appropriate. Continuous variables were reported as mean ± standard deviation (SD) and range. Categorical variables were presented as frequencies and percentages. Data collection was performed using Microsoft Excel (Microsoft Corporation, Redmond, WA) for Windows. Data analysis was accomplished with SPSS Statistics version 23 (IBM Corp. Released 2015. IBM SPSS Statistics for Windows, Version 23.0. IBM Corp. Armonk, NY, USA). Differences were assessed using the Fisher exact test for categorical variables and Mann–Whitney or Wilcoxon, when appropriate, for continuous variables. A *P* value less than 0.05 was considered statistically significant.

Effect size was calculated using the following indices: Cohen’s d, Cohen’s w, and odds ratio, in relation to the different types of data.

A post hoc power analysis was performed, assuming an alpha-type error of 0.05 and a power of at least 0.8, with an enrollment ratio of 0.2 and a minimal clinically significant difference of a 20% decrease in HHS after neurological complications. The minimum number of cases to be studied was determined to be 18 (15 VS 3). Therefore, the sample size was adequate to assess significant differences.

## Results

In a series of 1,450 anterior THA procedures performed from January 2018 to December 2021, six patients (0.4%) who underwent THA through a non-standard surgical interval were identified and studied. Data were presented at an average follow-up of 33 ± 22.3 months (range 9–72). *As per* inclusion criteria, no significant differences were found in terms of age, gender, and BMI compared to the control group of 50 standard DAA THA patients (Table 1).

**Table 1:** Patients’ demographics

	**Study group (*n* = 6)**	**Control group (*n* = 50)**	***P***	**Effect size**
Age at surgery	72.8 ± 11.1 (56–85)	75.3 ± 5.3 (43–87)	0.345^a^	0.287^c^
Sex (M-F)	3F (50%), 3 M (50%)	29F (58%), 21 M (42%)	0.708^b^	0.218^d^
BMI	30.5 ± 3.2 (26–35)	29.7 ± 2.1 (23–39)	0.409^a^	0.295^c^
Follow-up (months)	33.0 ± 22.3 (9–72)	30.0 ± 1.2 (30–32)	0.317^a^	0.189^c^
Medical history and comorbidities	Diabetes: 1 (17%) Smoker: 1 (17%) Cardiovascular diseases: 1 (17%) Obesity: 3 (50%)	Diabetes: 5 (10%) Smokers: 7 (14%) Cardiovascular diseases (including hypertension): 12 (24%) Obesity: 13 (26%) Renal failure: 1 (2%) Incontinance of urine: 7 (14%) Asthma or COPD: 6 (12%) Bowel disorder: 2 (4%)		

^a^Mann–Whitney test

^b^Fisher exact test

^c^Cohen’s d

^d^Cohen’s w

Surgical time, lasting from skin incision to skin closure, was significantly longer in the study group, with an average time of 91 ± 34.8 min (range, 52–139), and 72.3 ± 14.2 min (range, 36–101) in the controls (*P* = 0.0147). No differences in terms of pre- and postoperative hemoglobin levels were observed. HHS significantly improved after surgery in both groups (*P* > 0.001). When study and control groups were compared, no difference was observed preoperatively in the average HHS score, whereas postoperatively, HHS scores were significantly lower in the study group (*P* = 0.0023) at the last available follow-up (Table 2).

**Table 2:** Operative data, blood loss, clinical outcomes, and complications

	**Study group (*n* = 6)**	**Control group (*n* = 50)**	***P***	**Effect size**
Operative time (min)	91 ± 34.8 (52–139)	72.3 ± 14.2 (36–101)	**0.0147**^**a**^	**0.703**^**c**^
ΔHb	4.1 ± 1 (3–5.3)	3.9 ± 0.4 (2.5–5.5)	0.3467^a^	0.262^c^
HHS pre	52.3 ± 7.6 (44–62)	54.6 ± 4.8 (48–71)	0.3035^a^	0.361^c^
HHS post	88 ± 9.3 (75–100)	96 ± 5.3 (83–100)	**0.0023**^**a**^	**1.056**^**c**^
ΔHHS	35.7 ± 11.3 (18–47)	41.4 ± 5.1 (29–47)	**0.0352**^**a**^	**0.650**^**c**^
Neurological complications (No. patients)	*n* = 4 (66.7%): 3 Femoral nerve lesions 2 LFCN lesions	*n* = 2 (4%): 2 LFCN lesions	**0.0006**^**b**^	**48**^**d**^

Bold type: statistically significant (*P* < 0.05)

*LFCN* Lateral femoral cutaneous nerve

^a^Mann–Whitney test

^b^Fisher exact test

^c^Cohen’s d

^d^Odds ratio

Neurological complications were significantly higher in the study group, whose patients reported a higher frequency of intraoperative femoral nerve and lateral femoral cutaneous nerve neuroapraxia. Four out of six patients (66.7%) developed neurological deficits attributable to femoral nerve and/or LFCN damage. No differences in terms of other intra- and postoperative complications were reported (Table 2).

In the study group, in one out of 6 patients (16.7%), the interval used was medial to the sartorius and rectus femoris, while in five out of 6 (83.3%), the interval was lateral to the sartorius and medial to the rectus femoris. In three out of six patients (50%), the surgeon realized to be in the incorrect interval before femoral neck resection, in two cases (33%) during cup preparation and acetabular component placement, and in one patient at the end of the procedure. Operative data are detailed in Table 3.

**Table 3:** Detailed operative data, blood loss, Clinical outcomes and complications in the cases of the study group

Cases	Intermuscular interval	When the medial interval was realized	Surgical note	Operative time (min)	ΔHb	HHS pre	HHS post	ΔHHS	Neural Complications
1	medial to rectus femoris	Capsule exposure	Exploration of the femoral neurovascular bundle	127	4.8	57	87	30	Femoral nerve lesion (partially recovered MRC 4/5), LCFN lesion (unrecovered)
2	medial to rectus femoris	After definitive component reduction		52	3.2	48	94	46	LCFN lesion (unrecovered)
3	medial to sartorius	Acetabular reaming	Improper patient positioning (hip abducted and externally rotated	64	4.8	45	92	47	Not reported
4	medial to rectus femoris	Capsule exposure	Complete release of the origin of the rectus femoris and subsequent reattachment	139	5.3	62	80	18	Femoral nerve lesion (partially recovered MRC 3/5), LCFN lesion (unrecovered)
5	medial to rectus femoris	Cup placement		78	3.5	58	100	42	Not reported
6	medial to rectus femoris	Capsule exposure		86	3	44	75	31	Femoral nerve lesion (minimally recovered MRC 2/5)

*LFCN* Lateral femoral cutaneous nerve, *MRC* Medical Research Council

**Fig. 2:**
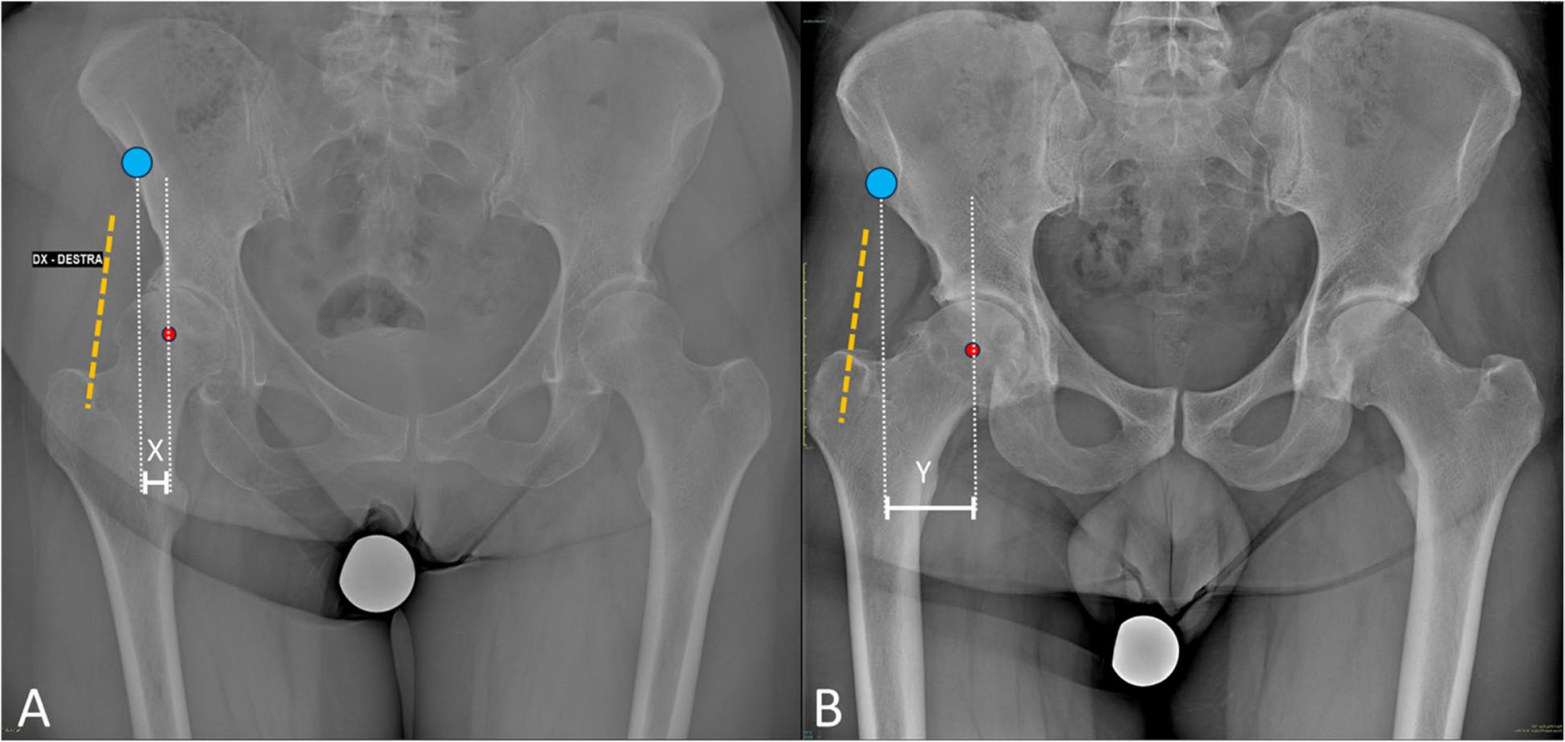
Radiograph A shows the preoperative radiograph of one of the cases receiving an excessively medial anterior approach is presented, where the horizontal distance between the ASIS (blue dot) and the center of rotation of the femoral head (red dot) is represented by distance X, compared to the same distance Y shown in radiograph B, which belongs to a patient in the control group. It is important to highlight that the surgical incision (outlined in orange) depends on the position of the ASIS, thus occasionally being more or less medial relative to the joint and the surrounding tissues

No significant differences were observed in terms of cup orientation and stem positioning on plain radiographs. By evaluating preoperative radiographs of the patients in the study group, an increased lateralization of the centre of rotation (COR) of the hip, compared to the anterior superior iliac spine (ASIS), was observed. To better quantify this parameter, in the absence of a validated radiographic index, the “ASIS-COR Distance” was calculated, consisting of the ratio between the horizontal distance between the ASIS and the COR of the hip, and the width of the pelvis on a coronal pelvis radiograph (Fig. 2). In the study group, this ratio was 0.11 ± 0.03 (range 0.92–0.141), being significantly lower compared to the control group, in which it averaged 0.135 ± 0.02 (range, 0.113–0.167) (*P* < 0.001).

Table 4 summarizes the number of previous DAA THA surgeries and average THA procedures per year for each surgeon at the time of the DAA THA through an unusual interval. Six out of six cases (100%) occurred during the first 100 DAA THAs performed by the surgeon [16–18].

**Table 4:** Data on anterior THA experience for each lead surgeon

Case #	Previous anterior THA	Number of DAA THA per year^a^
1	23	88
2	12	45
3	62	75
4	43	45
5	98	53
6	78	88

^a^considering the year in which the cases occurred

## Discussion

DAA approach to the hip can be rarely associated with excessive medial exposure to the joint, in particular, during the learning curve, defined as the first 100 surgeries [18]. A medial approach means longer operative time, increased risk of neurological complications, and inferior clinical outcomes, thereby rendering this event clinically significant. No demographic characteristics were associated with this occurrence. However, we outline an anatomic pattern of excessive lateralization of the COR of the hip compared to the ASIS which could represent a risk factor.

The described event was not reported in current literature, as there were no studies or descriptions in textbooks that detail this occurrence. However, the medialized approach to the hip may be one of the major determinants of neurological complications in DAA THA. Less experienced surgeons should be aware of this possible pitfall, which could account for potential neurological issues of femoral nerve or FCLS deficits.

Regarding intraoperative data, a significant increase in surgical time was observed in patients with too medial exposure of the hip joint, while no significant difference was found in blood loss. Surgical time is a very important factor, potentially responsible for an increased rate of infections [19] and other complications that can worsen clinical outcomes [12, 17]. In the study group, the increased operative time was likely attributable to the use of an unconventional approach, resulting in reduced surgeon confidence throughout all phases of the procedure because of the loss of anatomical landmarks.

Patients showed worse outcomes in terms of Harris Hip Score (HHS) at the last available follow-up of 33 months. These findings were potentially attributable to the higher incidence of neurological complications and, above all, to the deficits of the femoral nerve. Indeed, the worse clinical outcomes were reported by the 3 patients with femoral nerve deficits, suggesting a potential cause of litigation [12, 20].

Nerve complications involving the femoral nerve during DAA THA are rare, with incidences ranging from 0% to 5% [20–22]. This means a reduction in quadriceps muscle strength (up to paralysis). When LFCN is involved, a diminished or absent sensitivity at the anterior aspect of the thigh is expected [22]. LCFN deficit is more frequent in DAA THA, being reported in up to 81% of patients [20], although it may cause discomfort and is less disabling. An excessively medial exposure of the hip joint can be a significant contributor to the incidence of postoperative neurological complications [23, 24]. The wrong anterior intermuscular interval inevitably reduces the thickness of soft tissues protecting the femoral neurovascular bundle, which can be compressed or injured by retractors [25, 26]. Likewise, violation of the connective tissue surrounding the neurovascular bundle can trigger the formation of hematoma or swelling of the soft tissues, which can compress the neurovascular structures. Current findings support the assertion that a significant proportion of femoral nerve-related complications after anterior THA can be attributed to this technical pitfall, particularly when other potential causes, including excessive limb-lengthening, excessive hip external rotation, and hyperextension, inadequate anterior acetabular retractor placement, seem unlikely.

Considering the demographic characteristics of the patients (age, gender, and BMI), no significant differences were found between the study group and the control group, suggesting the absence of identifiable demographic risk factors. However, a particular radiographic characteristic was highlighted as a potential risk factor for an accidental medial approach to the hip joint, namely, the lateralization of the native hip COR in relation to the anterior superior iliac spine (ASIS). The ASIS is often used as a landmark for skin incision, and therefore, a radiographic and anatomical variant could lead to an alteration of the reference points for the subsequent surgical steps. Surgeons should be aware that reliance on standard landmarks for incision may be improper in the cases where the native anatomy deviates from the norm, including extruded femoral head or increased internal rotation of the iliac wings [27] (Fig. 2).

Taking into account the surgeon’s experience, available data suggest that, in all cases, surgeons were still in training for DAA. These findings reinforce the concept that DAA requires a long learning curve [18], currently defined as the first 100 cases, during which complications can occur, affecting the outcomes of the procedure. No cases were registered after the first 100 interventions by any single surgeon. Therefore, it can be determined that beyond this limit, inadvertent too medial anterior approach becomes negligible in clinical practice. For this reason, it is advisable to recommend that at least the first 50 to 100 surgical approaches are performed alongside a senior surgeon to avoid pitfalls.

Several tips could help reduce the occurrence of this complication, including adequate anatomic cadaver lab training, assistance from an experienced surgeon during the learning curve, and a standardized intraoperative routine characterized by reproducible steps and checkpoints to ensure correct positioning throughout each phase of the procedure. In this setting, the use of a positioning table could facilitate the standardization of each phase of the procedure, although it is a widely debated opinion in literature [28]. Patient positioning during the surgical approach is of paramount importance, whether a positioning table is used or not. The lower limb should be placed in slight flexion to relax the flexor muscles, and in internal rotation to keep the patella neutral and the surgical interval towards the surgeon. The limb should not be adducted or abducted, with the hip kept in a neutral position on the frontal plane.

The incision should be subject to careful consideration. It should not be overly small during the learning curve, and it should be performed lateral with respect to the TFL muscle. After subcutaneous tissue dissection, the aponeurosis of the TFL and the interval with the sartorius muscle must be appreciated and developed. The aponeurosis should be incised laterally over TFL muscle belly, and the interval developed from lateral to medial. When there is doubt regarding the correct identification of the muscle bellies, the palpation of anterior inferior iliac spine and the orientation of the muscle fibers from proximal to distal, directed laterally in the case of the TFL and medially for the sartorius, may help the surgeon to find the route. If the surgeon had any doubt about the route, a tip to follow is “never be medial to the ASIS”.

Upon reaching the deep plane of the interval, care must be taken to remain lateral to the rectus femoris muscle and not to go medially to the ASIS. The lateral circumflex femoral vessels (ascending branch) can be employed as a reliable landmark to confirm the correct interval [29–31]. These are indeed well identifiable laterally to the rectus femoris, in a very consistent position between the proximal two-thirds and the distal third of the incision. In case of a too medial approach, the tiny muscular branches of the femoral nerve (Fig. 1) may resemble the vessels and could disorient the surgeon. The distal or proximal extension of the incision is, in any case, justified in case of doubt to ensure better exposure and locate the circumflex vessels in a more distal position.

When realizing that the wrong interval has been entered, it is necessary to restart the exposure from the superficial interval and to identify the anatomic landmarks. These strategies help ensure the procedure is conducted as safely and efficiently as possible, whether or not a positioning table is used.

An excessively medial approach to the hip was a rare occurrence in our series, occurring in only 0.4% of the overall number of operated patients. However, given the incidence of neurological complications, it is a potentially severe condition that may compromise the clinical outcomes.

This study has several limitations. The retrospective nature of the research, coupled with the limited number of included patients, limited the robustness of the findings and their statistical significance. Furthermore, this study reflected the experience of a single high-volume centre for DAA THA, in which all surgeons use a standardized approach. Surgeons involved in the study displayed different levels of surgical skills, which were influenced by their years of service and the specific training experience. However, the main limitation of the study is the risk of underestimation of the error in the surgical approach, because of inadequate description of the event in the surgical records. However, we tried to compensate for the limitation by retrieving data from weekly grand round discussions for debriefing of the operated cases. On the other hand, it is possible that certain cases receiving unconventional approaches were not recognized and, therefore, were not included in this study. It is conceivable that in some instances, surgeons may not have recognized the use of a non-standard approach, attributing any complications to anatomical variations and severity of hip osteoarthritis.

## Conclusions

An excessively medial anterior approach is a rare but potentially serious pitfall while performing THA. This condition is not described in literature, and hence it may not be recognized and managed appropriately. An excessive medial exposure to the hip joint during DAA is related to prolonged operative time, decreased functional outcomes, and an increased occurrence of neurological complications affecting the femoral nerve or the LCNF. It has also been highlighted that such an event typically occurs within the first 100 THAs, confirming the significance of the learning curve for the DAA. To avoid unconventional intermuscular intervals, particular attention is paid to patients whose hip joint is lateralized relative to the anterior superior iliac spine. Additionally, correct patient positioning, recognition of muscle bellies, and identification of the circumflex vessels may prevent the surgeon from an inadvertent violation of the correct intermuscular interval.

This case series serves as a reminder of the potential complications arising from deviating from the standard anterior approach to the hip during THA surgery. Current report deserves recognition to support those new to DAA THA performance. Adequate training, anatomical education, and mentoring support during the learning curve of this surgery might improve surgeons’ confidence and adequately protect patients’ safety.

## Data Availability

Not applicable.

## Abbreviations

DAA: Direct Anterior Approach
THA: Total Hip Arthroplasty
TFL: Tensor Fasciae Latae
RF: Rectus Femoris
ERAS: Enhanced Recovery After Surgery
OA: Osteoarthritis
BMI: Body Mass Index
HHS: Harris Hip Score
ASIS: Anterior Superior Iliac Spine
LFCN: Lateral Femoral Cutaneous Nerve
COR: Center of Rotation
